# Insights from the reanalysis of high-throughput chemical genomics data for *Escherichia coli* K-12

**DOI:** 10.1093/g3journal/jkaa035

**Published:** 2020-12-22

**Authors:** Peter I-Fan Wu, Curtis Ross, Deborah A Siegele, James C Hu

**Affiliations:** 1 Department of Biochemistry and Biophysics, Texas A&M University and Texas Agrilife Research, College Station, TX 77843-2128, USA; 2 Department of Biology, Texas A&M University, College Station, TX 77843-3258, USA

**Keywords:** phenotypic profiling, functional genomics, microbial genomics, microbial genetics, high-throughput studies

## Abstract

Despite the demonstrated success of genome-wide genetic screens and chemical genomics studies at predicting functions for genes of unknown function or predicting new functions for well-characterized genes, their potential to provide insights into gene function has not been fully explored. We systematically reanalyzed a published high-throughput phenotypic dataset for the model Gram-negative bacterium *Escherichia coli* K-12. The availability of high-quality annotation sets allowed us to compare the power of different metrics for measuring phenotypic profile similarity to correctly infer gene function. We conclude that there is no single best method; the three metrics tested gave comparable results for most gene pairs. We also assessed how converting quantitative phenotypes to discrete, qualitative phenotypes affected the association between phenotype and function. Our results indicate that this approach may allow phenotypic data from different studies to be combined to produce a larger dataset that may reveal functional connections between genes not detected in individual studies.

## Introduction

Genome-wide genetic screens and chemical genomic studies, pioneered in yeast (Giaever and Nislow, 2014), are now widely used to study gene function in many model organisms, including the bacterium *Escherichia coli* (Nichols *et al.* 2011; Campos *et al.* 2018; Price *et al.* 2018). Based on the same principle that underlies the interpretation of forward genetic studies—that mutations that cause similar phenotypes are likely to affect the same biological process(es)—these high-throughput approaches have led to insights into the biology of a variety of organisms (Hillenmeyer *et al.* 2010; Arnoldo *et al.* 2014; Shefchek *et al.* 2020). It has been concluded that the collective phenotypic expression pattern of an organism can serve as a key to understand growth, fitness, development, and diseases (Bochner 2009; Houle *et al.* 2010).

Despite the demonstrated success of high-throughput phenotypic studies at predicting functions for genes of unknown function or predicting new functions for well-characterized genes, their potential to provide insights into gene function has not been fully explored. There does not seem to have been a systematic comparison of different metrics for measuring the similarity of phenotypic profiles. Further, while the likely benefits of combining information from high-throughput phenotypic studies from different laboratories have been recognized, very few methods of doing this have been described (Hoehndorf *et al.* 2013; Shefchek *et al.* 2020).

Here, we report reanalysis of the data from a published high-throughput phenotypic study of *Escherichia coli* K-12 (Nichols *et al.* 2011). *Escherichia coli* is one of the best-studied bacterial organisms, and the availability of high-quality annotation sets with information on gene function and regulation allowed us to compare the ability of different metrics for measuring phenotypic profile similarity to correctly infer gene function. We conclude that there is no single best method for comparing phenotypic profiles. Overall, the three metrics we tested gave comparable results for most gene pairs. However, there were instances where the metrics behaved differently from one another. We also assessed how converting quantitative phenotypes to discrete, qualitative phenotypes affected associations between phenotype and function. Our results indicate that this may be a viable approach for combining phenotypic data from different studies, creating a larger dataset that may reveal functional associations not detected by individual studies alone.

## Materials and methods

### Sources of data

The high-throughput phenotypic profiling data as normalized fitness scores were downloaded from Supplementary Table S2 of the original paper (Nichols *et al.* 2011). Missing values (0.17% of total fitness scores) were replaced with population mean as an imputation method. In Supplementary Table S2, fitness scores were associated with the relevant mutant gene with ECK identifiers. In order to map functional annotations to these genes, the ECK identifiers were verified, corrected, and mapped to b numbers and EcoCyc gene identifiers using information in the genes.dat file from EcoCyc release 21.0. This and other EcoCyc files were downloaded from their website (https://biocyc.org/download.shtml).

The six annotation sets were obtained from various sources. EcoCyc pathway annotations were mapped to each gene using information in the pathways.col file (EcoCyc release 21.0). EcoCyc protein complex annotations were mapped to each gene using information in the protcplxs.col file (EcoCyc release 21.1) after removal of homomeric protein complexes. KEGG module annotations were obtained and mapped by retrieving module name and b numbers from the KEGG website (https://www.kegg.jp). Operon annotations were mapped to each gene using information in the id_mapping/transunits.dat file (EcoCyc release 21.1). Regulon annotations were obtained and mapped to each gene using a download of Regulon DB version 9.4 (http://regulondb.ccg.unam.mx). The object_synonym.txt file was used to map ECK12 gene identifiers to ECK gene identifiers. RegulonDB annotations were then obtained from the file regulon_d_tmp.txt and mapped to ECK identifiers.GO biological process annotations were obtained from the Ecocyc gene_association.ecocyc file (EcoCyc release 21.1) and mapped to each gene to produce the file 2017_05_ECgene_association.ecocyc.csv. UniProt IDs retrieved from the Bioconductor package UniProt.ws were used to associate GO annotations from proteins to genes. The annotation sets, the number of genes annotated by each annotation set, and the total number of annotations are summarized in Table 1.

**Table 1:** Sources of the gene annotations used in this study

Annotation set (source)	**Number of annotated genes** ^a^	**Total number of gene annotations** ^b^
1. EcoCyc pathways (EcoCyc)	885	2,317
2. Heteromeric protein complexes (EcoCyc)^c^	688	871
3. Operons (RegulonDB)	3,858	5,349
4. Regulons (RegulonDB)	1,572	3,886
5. Modules (KEGG)	333	524
6. GO biological process annotations	2,609	5,775
7. Annotation to both EcoCyc pathways and heteromeric protein complexes (intersection of annotation sets 1 and 2)	188	818^d^
8. Annotation in each of annotation sets 1–5 (intersection of annotation sets 1–5)	77	922^e^
9. Annotation to either EcoCyc pathways or heteromeric protein complexes (union of annotation sets 1 and 2)	1,385	3,269
10. Annotation in any of annotation sets 1–5 (union of annotation sets 1–5)	3,866	12,937

a Number of annotated genes that were deleted or otherwise mutated in the set of strains used in the original study (Nichols *et al.* 2011).

b Total number of annotations associated with the genes in the first column.

c We have excluded genes annotated to EcoCyc protein complexes that are homomeric complexes.

d This is the number of annotations associated with any of the 188 genes that are annotated to both annotation sets.

e This is the number of annotations associated with any of the 77 genes that are annotated in each of annotation sets 1–5.

### Statistical analysis and software

The statistical programming language R was used throughout the study. Phenotypic profile similarity was calculated using Pearson correlation coefficient (|PCC|), Spearman’s rank correlation coefficient (|SRCC|), mutual information (MI), and semantic similarity. PCC and SRCC were calculated using the cor() function, with the metric argument specified by either “pearson” or “spearman.” Different implementations are needed to calculate MI for continuous, quantitative data and discretized, qualitative data. MI for quantitative data was calculated using the cminjk() function provided in the mpmi package (https://cran.r-project.org/web/packages/mpmi/index.html), while MI for discretized data was calculated using the mutinformation() function provided in the infotheo package (https://CRAN.R-project.org/package=infotheo). For the plots of precision versus ranking based on phenotypic profile similarity (Figures 2–4 and 6), the negative control is precision calculated for randomly ordered gene pairs that were generated using the R function sample() to permute the rankings of all possible gene pairs. For precision-recall curves (Supplementary Figures S6-S8), the negative control is precision calculated for 5000 gene pairs selected randomly without replacement from the set of all possible gene pairs using the R function sample(). For all negative controls, the number of co-annotated gene pairs present in the set of all possible gene pairs differed depending on which annotation set or combination of annotation sets was used to identify co-annotated gene pairs, except Figure 2, where only the negative control using the union of annotation sets 1–5 is shown.

The semantic similarity of GO biological process annotations was calculated using a graph-based method (Wang *et al.* 2007). Calculations were performed using the GOSemSim package (Yu *et al.* 2010) from Bioconductor. For the Mann–Whitney *U* test, wilcox.test() function was used.

For violin plots, geom_violin() was used to plot the kernel density plot and geom_box() was used for the boxplot. Both functions are from the ggplot2 package (Wickham 2016). In the box plot associated with each violin plot, the middle line in the box represents the median; the whiskers indicate the 1.5 interquartile range away from either Q1 (lower box boundary) or Q3 (upper box boundary). For the violin plots that display the distribution of MI values for gene pair profile similarity determined using discretized, ternary fitness scores (Figures 5, A and B), the MI values were log transformed after addition of a constant (1 × 10^−6^) to eliminate zero values.

For each pathway and protein complex in Supplementary Figures S1 and S2, a permutation-based *P*-value was calculated by randomly sampling the same number of phenotypic profiles as the number of genes contained in each pathway or protein complex, calculating the mean pairwise profile similarity based on |PCC|, repeating 1000 times, and then calculating the fraction of these mean |PCC| values that has a higher mean |PCC| than the actual |PCC| value for that pathway or protein complex.

### Data availability

The code and data files used for calculations and reproducing the results are available on GitHub: https://github.com/peterwu19881230/Systematic-analyses-ecoli-phenotypes.

Supplementary material is available at figshare DOI: https://doi.org/10.25387/g3.13350674.

## Results

### Phenotypic profiles and the functional annotation sets used

We start with descriptions of the phenotype data and functional annotation sets that were used for our analysis. The phenotypic profiles come from a high-throughput chemical genomics study of *E. coli* K-12 (Nichols *et al.* 2011). Growth phenotypes for 3979 mutant strains, which were primarily single-gene deletions of nonessential genes, were based on sizes of spot colonies grown under 324 conditions, which represented 114 unique stresses. For each of the growth conditions, fitness scores were obtained and scaled to a standard normal distribution. Positive scores indicate increased fitness and negative scores indicate decreased fitness.

Six annotation sets were used as sources of information about gene function. The number of genes annotated in each annotation set and the total number of annotations for each annotation set are shown in Table 1. Annotations of *E. coli* genes to metabolic and signaling transduction pathways (annotation set 1) and to heteromeric protein complexes (annotation set 2) were obtained from EcoCyc (Keseler *et al.* 2017); annotation of genes to operons (annotation set 3) and to regulons (annotation set 4) were extracted from EcoCyc and RegulonDB (Gama-Castro *et al.* 2016); and annotations of genes to KEGG modules (annotation set 5), which associate genes to metabolic pathways, molecular complexes, and also to phenotypic groups, such as pathogenesis or drug resistance, were obtained from the Kyoto Encyclopedia of Genes and Genomes (KEGG) (Kanehisa *et al.* 2016). For these five annotation sets, genes were scored as co-annotated if they shared the same annotation(s) from one or more of the annotation sets, for example, being annotated to the same pathway or protein complex, *etc*.

The annotations of *E. coli* genes with Gene Ontology (GO) biological process terms (annotation set 6) (Gene Ontology Consortium 2017) were obtained from EcoCyc. The GO biological process annotations of *E. coli* genes were treated separately from the other five annotation sets because GO’s directed-acyclic graph structure allows semantic similarity rather than co-annotation to be used for assessing functional similarity (Pesquita 2017). Simply looking for co-annotations with the same GO term(s) will include co-annotations to high-level terms, such as “GO:0044237 cellular metabolic process” or “GO:0051716 cellular response to stimulus,” terms that do not provide very specific information about function. Also, co-annotations would not capture instances where two genes are annotated with related, but not identical, terms. These limitations can be overcome by using semantic similarity rather than co-annotation to estimate functional similarity from GO annotations. The method for determining the semantic similarity of two GO terms developed by Wang *et al.* (2007) takes into account the locations of the terms in the GO graph, as well as incorporating the different semantic contributions that a shared ancestral term may make to the two terms, based on the logical relationship, such as “is_a” or “part_of,” that connect the term to the shared ancestor. In addition, when calculating functional similarity, the Wang method includes both identical GO terms and semantically similar GO terms associated with the two genes being compared.

### Functional connections between genes enriched for higher phenotypic profile similarity

The association between phenotypic profiles and functional annotations was examined from two perspectives: First, are gene pairs that share the same annotation(s), that is, co-annotated gene pairs, more likely to have higher phenotypic profile similarity? Second, are gene pairs with higher phenotypic profile similarity more likely to be co-annotated?

**Figure 1:**
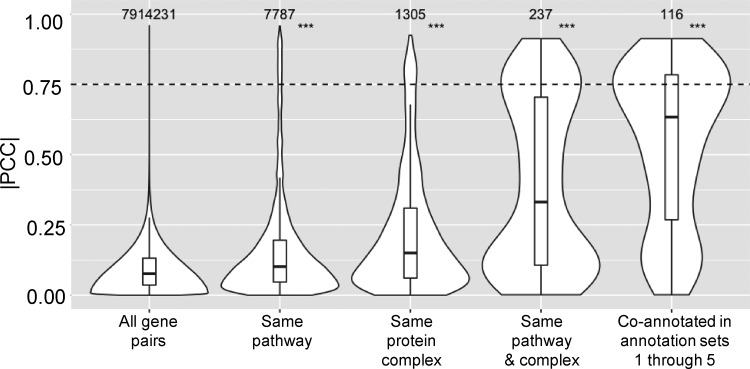
Higher phenotypic similarity was found for co-annotated gene pairs. Violin plots of the distributions of |PCC| values for, from left to right, all possible gene pairs, gene pairs annotated to the same EcoCyc pathway, gene pairs annotated to the same heteromeric protein complex, gene pairs annotated to the same EcoCyc pathway and heteromeric protein complex, and gene pairs that are co-annotated in annotation sets 1 through 5 (the intersection of EcoCyc pathways, heteromeric protein complexes, operon, regulon, and KEGG module). Numbers above each violin plot indicate the number of gene pairs in each plot. ***: *P*-value < 0.001 was determined by 1-sided Mann-Whitney *U* test, compared to all gene pairs. The dashed line indicates |PCC| = 0.75, which was chosen as an arbitrary cut-off.

To address whether co-annotated gene pairs have higher phenotypic profile similarity, we used PCC to assess the phenotypic profile similarity. This metric was chosen because it is probably the most widely used metric to assess phenotypic profile similarity and was the metric used in the original paper for comparing phenotypic profiles (Nichols *et al.* 2011). To visualize the results, the distributions of the absolute value of PCC (|PCC|) for gene pairs were plotted as violin plots for various combinations of annotation sets (Figure 1). The first violin plot shows the distribution of |PCC| values for all possible gene pairs (mean |PCC| = 0.09). The majority have a |PCC| value < 0.25 and only 0.16% have a |PCC| value >0.75 (an arbitrarily chosen cut-off based on Hinkle *et al.* 2003). When only gene pairs that are co-annotated to the same EcoCyc pathway were considered (second violin plot), there was a statistically significant increase in the mean |PCC| value (0.16), and the percentage of gene pairs with |PCC| > 0.75 increased 20-fold. Similar results were seen for gene pairs that are co-annotated to the same heteromeric protein complex (third violin plot, mean |PCC| = 0.22). When considering only gene pairs that are co-annotated to more than one annotation set (fourth and fifth violin plots), even higher phenotypic profile similarity was observed (mean |PCC| = 0.39, 0.54, respectively), supporting the expectation that gene pairs with stronger functional associations will have more similar phenotypic profiles. The trend of there being a higher fraction of gene pairs with |PCC|>0.75 as functional associations increased also continued; this fraction increased from 0.16% for all gene pairs, to 3.2% for gene pairs in the same EcoCyc pathways, to 4.9% for gene pairs in the same heteromeric protein complexes, to 19% for gene pairs in the same EcoCyc pathways and heteromeric protein complexes, and to 30% for gene pairs that are co-annotated in annotation sets 1 through 5 (the union of EcoCyc pathways, heteromeric protein complexes, operons, regulons, and KEGG modules).

A more detailed analysis within the EcoCyc pathway or heteromeric protein complex annotations was conducted by examining all pairwise combinations of gene pairs within pathways or protein complexes that contain two or more gene products. Supplementary Figures S1 and S2 show the distributions of |PCC| values for all pairwise combinations of genes in each pathway or protein complex. For 70% of the pathways and 67% of the protein complexes analyzed the average |PCC| value is significantly higher than random expectation (|PCC| = 0.09).

### Phenotypic profile similarity is explained by functional annotations

**Figure 2:**
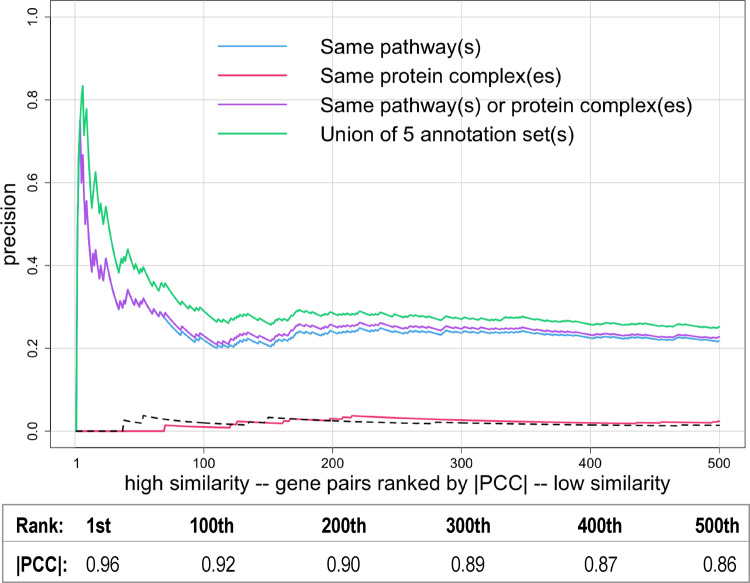
Increased co-annotation was found for gene pairs with higher phenotypic profile similarity. Gene pairs were ranked from high to low similarity based on |PCC| values and plotted versus precision, which was calculated as described in the text (only the first 500 gene pairs are shown). The different colored lines indicate either gene pairs that are annotated to the same EcoCyc pathway (blue), to the same heteromeric protein complex (pink), to either the same EcoCyc pathway or protein complex (purple), or are co-annotated in any of annotation sets 1 through 5 (the union of EcoCyc pathways, heteromeric protein complexes, operon, regulon, and KEGG module). Note that for the first few gene pairs the lines overlap, except the line for protein complexes. The dashed line shows precision for randomly ordered gene pairs generated as described in the Methods (negative control). The correspondence between ranking and |PCC| is shown below the graph.

To address the second question, which is whether gene pairs with higher phenotypic profile similarity are more likely to be co-annotated, we ranked gene pairs based on phenotypic profile similarity and then calculated precision based on whether or not gene pairs are co-annotated (Figure 2). Precision is the fraction of results that a test identifies as positive that represent true positives. Mathematically, precision, also known as the positive predictive value, is the number of True Positives divided by True Positives plus False Positives, or TP/(TP + FP). After ranking gene pairs based on phenotypic profile similarity expressed as |PCC| values, precision for each position *n* in the ranking was calculated considering gene pairs ranked at or above position *n* to be TPs if they are co-annotated or FPs if they are not co-annotated. For example, for the 100th gene pair in the ranking, precision is calculated for gene pairs 1 through 100. Figure 2 shows the plots of precision versus ranking for the top-ranking 500 gene pairs computed for single annotation sets or combinations of annotation sets. For gene pairs co-annotated to the same pathway(s), precision started at zero, because the highest ranked gene pair was not co-annotated, but then increased to ∼0.8 before gradually declining and leveling off at approximately 0.2. Surprisingly, for gene pairs co-annotated to the same protein complex, precision was very low and not significantly different from the precision values computed for randomly ordered gene pairs. Combining the annotation sets for pathways and protein complexes, brought a slight increase in precision. When operon, regulon, and KEGG modules were also included to define the broadest set of co-annotations, precision increased dramatically.

### The Pearson correlation coefficient is sensitive to the extreme fitness scores on minimal media

**Figure 3:**
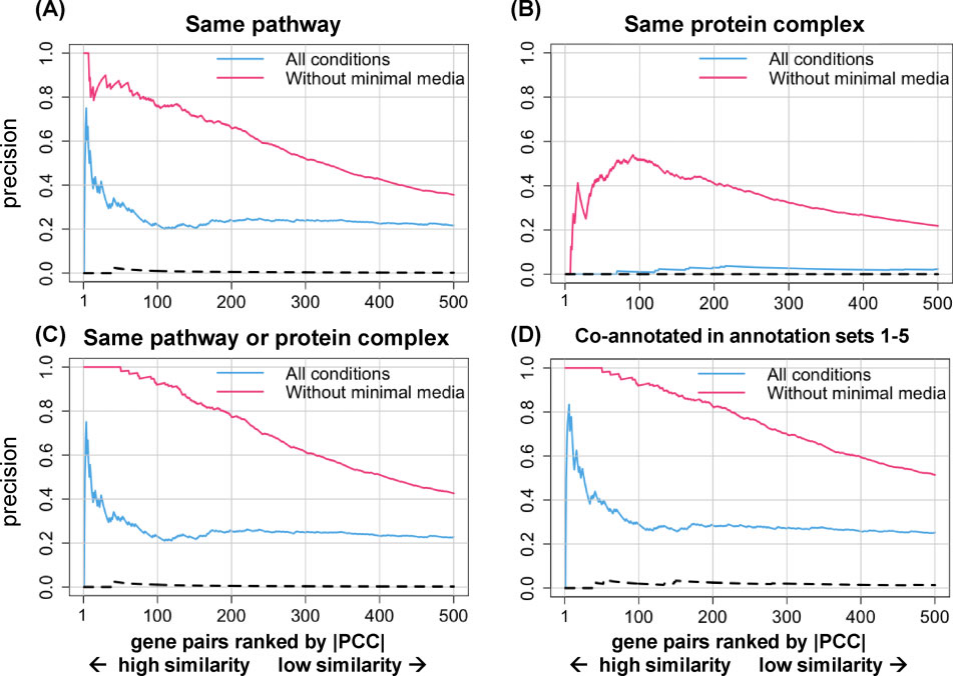
Precision increased when minimal media conditions were excluded. Gene pairs were ranked from high to low similarity based on |PCC| and plotted versus precision, calculated as described in the text (only the first 500 gene pairs are shown). The four panels show (A) gene pairs annotated to the same EcoCyc pathway, (B) gene pairs annotated to the same heteromeric protein complex, (C) gene pairs annotated to either the same EcoCyc pathway or protein complex, and (D) gene pairs co-annotated in any of annotation sets 1 through 5. The dashed lines show precision for randomly ordered gene pairs generated as described in the Methods (negative control). The correspondence between ranking and |PCC| is the same as in Figure 2.

To try to understand why precision was so low for protein complex annotations (Figure 2), we inspected the gene pairs and saw that 98 of the 100 top-ranking gene pairs consisted of genes coding for biosynthetic enzymes, and, in 84 of these 98 gene pairs, the genes were annotated to different biosynthetic pathways. For example, the top-ranked gene pair (|PCC| = 0.96) contained the genes *ilvC* and *argB*, which encode enzymes required for isoleucine-valine and arginine biosynthesis, respectively. Mutant strains lacking any of these biosynthetic genes would be auxotrophs and share the phenotype of little or no growth on unsupplemented minimal media. To test whether the |PCC|-based measure of phenotypic profile similarity was dominated by the large negative fitness scores associated with growth of auxotrophic mutants on minimal media, we excluded the fitness scores for the growth conditions that involved minimal media (10 out of 324 total conditions) and reassessed the relationship between precision and phenotypic profile similarity. As shown in Figure 3, even though only a small fraction of conditions was excluded, this change resulted in dramatically higher precision overall, not only for gene-pairs co-annotated to heteromeric protein complexes but also for gene-pairs co-annotated to either EcoCyc pathways, the union of EcoCyc pathways and heteromeric protein complexes, or the union of annotation sets 1 through 5. In addition, when strains known to have auxotrophic phenotypes were excluded from the analysis, little difference in precision was seen whether growth conditions involving minimal media were included or excluded (Supplementary Figure S3).

### Alternative metrics for measuring phenotypic profile similarity

There are other methods, besides the PCC, that can be used to assess phenotypic profile similarity. We chose the absolute value of |SRCC| or MI, which were implemented as described in the Methods, to measure similarity, and used the union of annotation sets 1 through 5 to score co-annotation. Violin plots of the distributions of phenotypic profile similarity obtained using these alternative metrics were not significantly different from the distributions seen using |PCC| as the metric (results not shown). In contrast, as shown in Figure 4A, the correlation between phenotypic profile similarity and precision was dramatically higher for |SRCC| and MI compared to |PCC|. For both |SRCC| and MI, precision was >0.9 for the top 100 ranked gene pairs and remained >0.5 for approximately the top 500 pairs. This result suggests that determining phenotypic profile similarity using SRCC or MI is less sensitive to the presence of a relatively small number of extreme phenotype scores than using the PCC, at least for this phenotypic dataset. If we recalculate precision for all three metrics after excluding the 10 growth conditions where auxotrophic mutants don’t grow, we see very little change in precision for gene-pairs ranked based on |SRCC| or |MI| (compare Figure 4, A and B). There is now very little difference in precision for the three metrics (Figure 4B). In addition, we calculated precision after removing the strains known to have an auxotrophic phenotype (Supplementary Figure S4). This result is consistent with Figure 4B in that all three metrics have similar precision.

**Figure 4:**
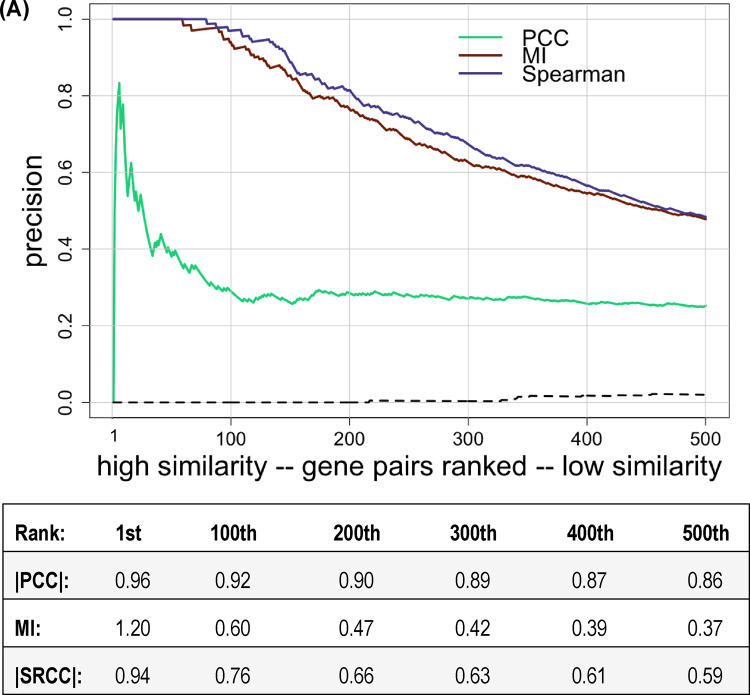
Precision versus ranking when different metrics are used to measure phenotypic profile similarity. Gene pairs were ranked from high to low similarity determined using either |PCC|, MI, or |SRCC| and plotted versus precision, using the union of annotation sets 1 through 5 to identify co-annotated gene pairs. Only the first 500 gene pairs are shown. Phenotypic profile similarity was assessed using either (A) all growth conditions or (B) excluding growth conditions with minimal media. The dashed line shows precision for randomly ordered gene pairs generated as described in the Methods (negative control). The correspondence between ranking and similarity scores is shown below each graph.

**Figure 4:**
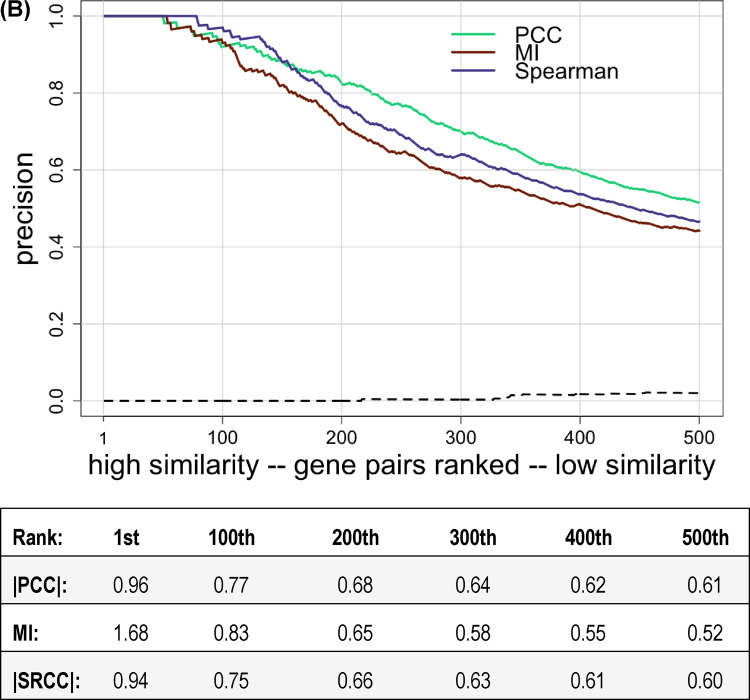
Continued.

### Simplified phenotypic profiles preserve biological meanings

Combining phenotypic information from different studies is expected to increase the likelihood of finding associations between genes and functions. However, the ability to combine datasets can be limited by differences in how quantitative phenotypes are scored in different studies. In addition, there is a need for methods to incorporate qualitative phenotypes, such as changes in cell or colony morphology, which are inherently qualitative, as well as changes in phenotype that are reported in a qualitative way, such as increased or decreased growth rate or increased or decreased resistance to a chemical. To address both of these issues we took the approach of converting quantitative phenotypes to qualitative phenotypes. We chose this approach because, if successful, it would allow a larger number of datasets to be combined. It would also allow us to utilize microbial phenotype information that has been collected and annotated with qualitative phenotype ontology terms in databases such as PomBase (Harris *et al.* 2013), SGD (Cherry *et al.* 2012), and OMP (Chibucos *et al.* 2014; Siegele *et al*. 2019).

**Figure 5:**
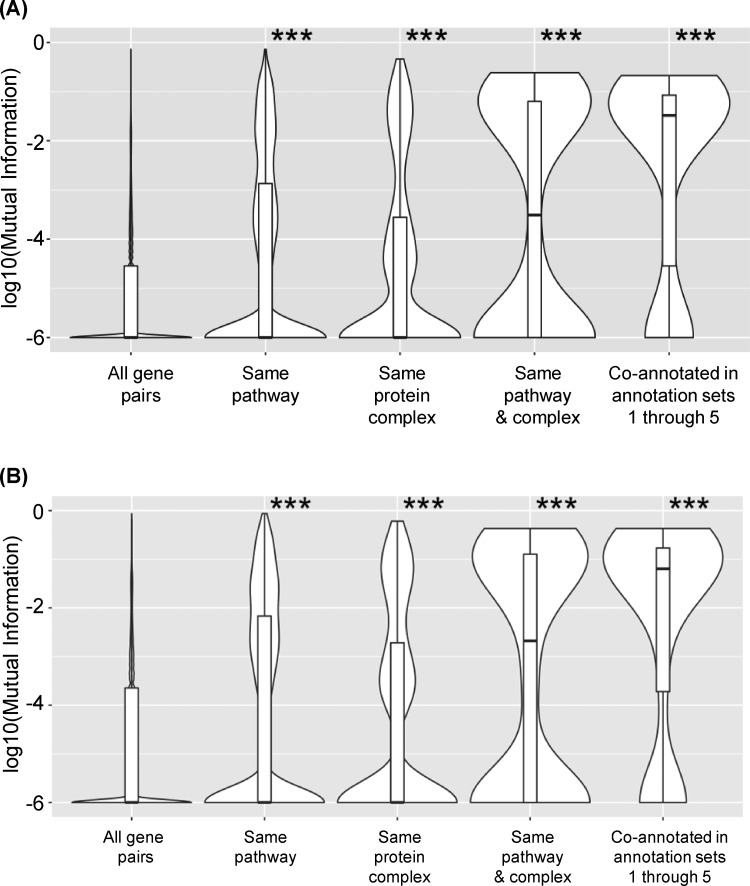
Phenotypic profile similarity after converting fitness scores from quantitative to qualitative, ternary values. Violin plots of the distributions of phenotypic profile similarity based on MI for, from left to right, all gene pairs, gene pairs annotated to the same EcoCyc pathway, gene pairs annotated to the same heteromeric protein complex, gene pairs annotated to the same EcoCyc pathway and heteromeric protein complex, and gene pairs that are co-annotated in annotation sets 1 through 5. The MI values were log transformed after addition of a constant (1 × 10^−6^) to eliminate zero values. The middle line within the box plots represents the median. Panel (A) shows the results when profile similarity was determined using all 324 growth conditions. The mean values of the distributions in (A) are 0.0006, 0.014, 0.014, 0.039, and 0.057. Panel (B) shows the results when profile similarity was determined after collapsing the growth conditions to 114 unique stresses. The mean values of the distributions in (B) are 0.0021, 0.026, 0.025, 0.073, and 0.1. ***: *P*-value < 0.001 determined by 1-sided Mann–Whitney *U* test, compared to all gene pairs.

The quantitative fitness scores in the phenotypic dataset were discretized to create a qualitative dataset with the fitness scores converted to 1, 0, or −1, where 1 stands for increased fitness, −1 for decreased fitness, and 0 for no difference in fitness compared to the mean fitness for all strains in a particular growth condition. The |PCC| cut-offs used to separate the quantitative fitness scores into discretized, ternary bins were based on the 5% false discovery rate (FDR) for each growth condition, which was the cut-off used to identify significant phenotypes in the original study (Nichols *et al.* 2011). Because the majority of strains have no significant phenotype in the growth conditions used (Nichols *et al.* 2011), after discretizing the data the majority of strains will have fitness scores of 0. Therefore, the PCC was no longer suitable for measuring phenotypic profile similarity. Instead, MI (Priness *et al.* 2007) was used as the scoring metric. The distribution of MI values for gene pairs were plotted as violin plots, after addition of a constant (1 × 10^−6^) to eliminate zero values followed by log transformation of the data. The first violin plot in Figure 5A shows the distribution of MI values for all possible gene pairs, followed by, from left to right, the distribution of MI values for gene pairs co-annotated to either the same EcoCyc pathway; the same heteromeric protein complex; to both an EcoCyc pathway and a heteromeric protein complex; or are co-annotated to the same EcoCyc pathway, heteromeric protein complex, operon, regulon, and KEGG module. As was seen for the mean |PCC| values in the analysis of the quantitative data (Figure 1), the mean MI values increased as the functional associations for a given gene pair increased (Figure 5A).

Another complication that can arise when trying to combine phenotype information from different studies is variation in the conditions used. For example, different studies may look at the effects of the same chemical but use different concentrations. To determine how removing concentration information affects phenotypic profile similarity, we reduced the original 324 growth conditions to 114 unique stresses. When different concentrations of a chemical were tested, for each strain only the concentration with the most significant fitness score was included and assigned a value of 1 or −1, as appropriate, or a score of 0 if no significant phenotype was seen for that treatment. The violin plots in Figure 5B show the distribution of MI values (after log transformation as described above) for all gene pairs and for different annotation sets or combinations of annotation sets for the reduced set of conditions. As seen for the full qualitative dataset, the mean MI values for co-annotated gene pairs in the reduced dataset were significantly higher than the mean MI value for all possible gene pairs (Figure 5B). In addition, when the distributions of gene pairs in the same co-annotation group are compared between Figure 5, A and B, significant differences of the means were observed for every co-annotated group (*P*-value < 0.001). Overall, these results indicate that useful inferences about gene function can still be made after the conversion of quantitative phenotypes to qualitative phenotypes and even after collapsing the number of phenotypes for each chemical treatment.

**Figure 6:**
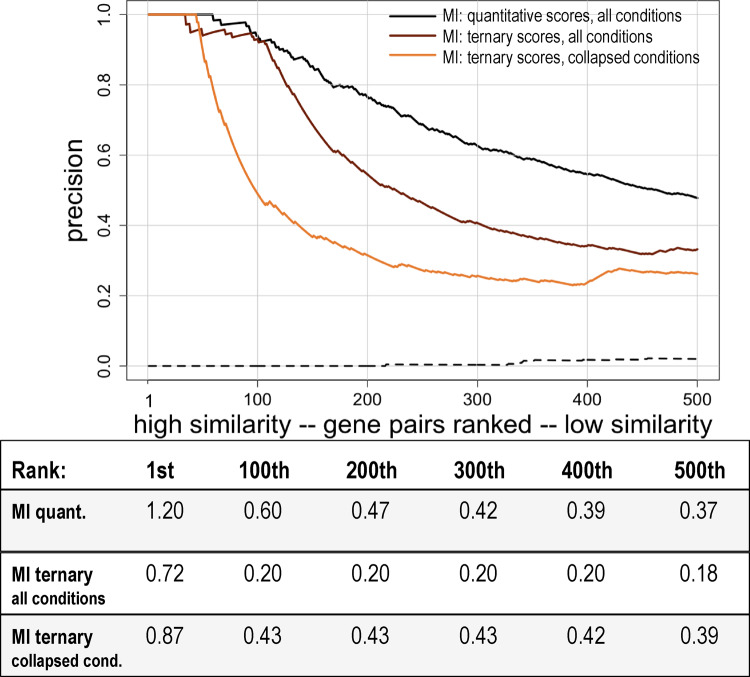
Precision versus ranking for quantitative versus discretized, ternary fitness scores. Gene pairs were ranked from high to low similarity based on MI and plotted versus precision using the union of annotation sets 1 through 5 to identify co-annotated gene pairs. Only the first 500 gene pairs are shown. Phenotypic profile similarity was determined with either the original quantitative fitness scores (black line), the discretized ternary scores for all growth conditions (brown line), or the discretized, ternary scores for growth conditions collapsed to 114 unique stresses (orange line). The cut-offs used to convert the quantitative scores to discretized, ternary scores were based on the 5% FDR for each condition. The dashed line shows precision for randomly ordered gene pairs generated as described in the Methods (negative control). The correspondence between ranking and similarity scores is shown below the graph.

We expected loss of information after converting quantitative phenotype scores to discretized, ternary fitness scores. To compare how many functional associations could still be retrieved using the qualitative scores, gene pairs were sorted based on their MI values determined using either quantitative phenotype scores, the qualitative ternary fitness scores, or the qualitative ternary fitness scores for the reduced set of conditions. Precision was then calculated, as described earlier, and was plotted versus ranking. As can be seen in Figure 6, precision is comparable for the top 100 gene pairs for both quantitative and for discretized, qualitative fitness scores. After this point, precision drops more quickly for the qualitative data than for the quantitative data. When precision for the reduced set of conditions is compared to precision for either of the other datasets, we see that precision drops off sooner and decreases more rapidly. Yet, precision is still much higher than for randomly ordered gene pairs, which indicates that functional associations can still be identified when qualitative, discretized fitness scores are used.

### Semantic similarity of GO annotations increased for gene pairs with shared functional annotations and with higher phenotypic profile similarity

**Figure 7:**
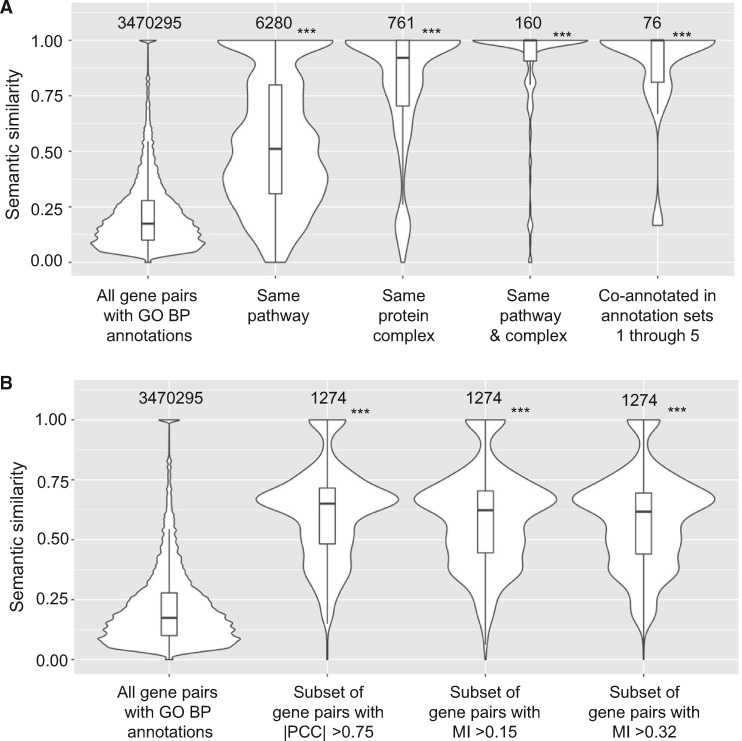
Higher semantic similarity and phenotypic profile similarity were found for co-annotated gene pairs. (A) Violin plots of the distributions of semantic similarity for, from left to right, all gene pairs annotated with GO biological process term(s), gene pairs annotated to the same EcoCyc pathway, gene pairs annotated to the same heteromeric protein complex, gene pairs annotated to both the same EcoCyc pathway and the same heteromeric protein complex, and gene pairs co-annotated in annotation sets 1 through 5. Numbers above each violin plot indicate the number of gene pairs in each plot. (B) Violin plots of semantic similarity for, from left to right, all gene pairs annotated with GO biological process term(s), the subset of gene pairs with |PCC|>0.75, the subset of gene pairs with MI >0.15 (calculated based on qualitative fitness scores for all growth conditions), and the subset of gene pairs with MI >0.32 (calculated based on qualitative fitness scores for the collapsed set of growth conditions). The cut-offs of MI >0.15 for the third violin plot and MI >0.32 for the fourth violin plot were chosen so that all three subsets of gene pairs would contain the same number of top-ranked gene pairs. ***: *P*-value <0.001 was determined by 1-sided Mann–Whitney *U* test, compared to all gene pairs.

Another way to assess whether two genes are likely to have similar functions is to compare the semantic similarity of the GO terms annotated to each gene. In the dataset from Nichols *et al.*, 66% (2609 out of 3979) of the strains used have mutations of genes that are annotated with GO biological process terms, which seemed a sufficient number to justify using this approach. Semantic similarity was computed using the method described by Wang *et al.* (2007), and the distribution of semantic similarity scores for all gene pairs where both members of the pair are annotated with at least one GO biological process term was compared to the distributions for subsets of gene pairs that have similar functions based on being co-annotated in one or more of the non-GO annotation sets. As shown in Figure 7A, semantic similarity increased when only co-annotated gene pairs were considered. The mean pairwise semantic similarity increased from 0.22 for all genes with GO biological process annotations (first violin plot), to 0.54 for gene pairs co-annotated to the same EcoCyc pathway (second violin plot), and to 0.80 for gene pairs co-annotated to the same heteromeric protein complex (third violin plot). Mean profile similarity was even higher for gene pairs that are co-annotated to both pathways and heteromeric protein complexes (mean = 0.90) as well as for gene pairs that are co-annotated in annotation sets 1–5 (mean = 0.89), as shown in the fourth and fifth violin plots, respectively. These results show that co-annotated gene pairs are also enriched for functional similarity based on GO biological process annotations.

To test whether gene pairs that have higher phenotypic profile similarity are more likely to have similar functions based on GO biological process annotations, we compared the distributions of semantic similarity values for all gene pairs annotated with GO biological process terms and for subsets of these gene pairs that have high phenotypic profile similarity based on |PCC| or MI. A cut-off of |PCC|>0.75 for the second violin plot was chosen arbitrarily to represent a moderate to high correlation (Hinkle *et al.* 2003), while the cut-offs of MI > 0.15 and >0.32 for the third and fourth violin plots, respectively, were chosen so that all three subsets of gene pairs would contain the same number (∼1200) of gene pairs. Comparison of the first two violin plots in Figure 7B shows that semantic similarity increased significantly for gene pairs with |PCC|>0.75 (mean semantic similarity = 0.61) compared to all gene pairs with GO biological process annotations (mean = 0.22). Enrichment for higher semantic similarity was also seen when phenotypic profile similarity was determined using discretized, ternary fitness scores either for all growth conditions (third violin plot, MI > 0.15, mean = 0.59) or for the collapsed set of 114 growth conditions (fourth violin plot, MI > 0.32, mean = 0.58). These results are consistent with those in Figure 1, which show higher phenotypic profile similarity for co-annotated gene pairs.

## Discussion

We systematically reanalyzed a published high-throughput phenotypic profile dataset for the model Gram-negative bacterium *E. coli* comparing different metrics for measuring phenotypic profile similarity, and assessing the effect of converting quantitative fitness scores to qualitative fitness on measurements of phenotypic profile similarity. We re-examined the *E. coli* phenotypic profiles in a pairwise fashion with the help of existing functional annotations. Overall, we found that gene pairs with functional associations are enriched for phenotypic profile similarity and that gene pairs with high phenotypic similarity scores tend to have functional associations.

Six high-quality annotations sets were used as sources of functional information. The gene annotations in EcoCyc, RegulonDB, KEGG, and GO come primarily from expert manual curation (Karp *et al*. 2018; Keseler *et al.* 2014, 2017; Gama-Castro *et al.* 2016; Kanehisa *et al.* 2016; Gene Ontology Consortium 2017). The GO biological process annotations include ∼1200 annotations (21%) that are inferred from electronic annotation without additional human review. We decided to include the electronic annotations in our analysis because most of them come from the transfer of annotations from orthologous gene products or are based on mappings from external sources, such as InterPro2GO or EC2GO, which have been shown to be very accurate (Hill *et al.* 2001; Camon *et al.* 2005; Holliday *et al.* 2017). Indeed, no significant difference was found in the semantic similarity of gene pairs whether electronic annotations were included (Figure 7B) or excluded (Supplementary Figure S5).

One aim of this study was to determine whether different metrics for determining phenotypic profile similarity differed in their ability to identify gene pairs with functional similarity. Comparison of the profile similarity scores for the top-ranked gene pairs showed that the three metrics used, |PCC|, |SRCC|, and MI, produced comparable results for most, although not all, gene pairs (data not shown). A more quantitative way to compare the performance of the metrics is by introducing precision: the fraction of positive results that are true positives. Gene pairs with phenotypic profile similarity above a specified cut-off were considered as positive results, and true positives were defined as gene pairs that are co-annotated in at least one of annotation sets 1–5. We chose to use precision rather than accuracy, which is the fraction of correct results, because the co-annotated and non-co-annotated gene pairs constitute a highly imbalanced dataset (Saito and Rehmsmeier 2015). Since the number of non-co-annotated gene pairs is much larger than the number of co-annotated gene pairs, high accuracy could be achieved by classifying all gene pairs as true negatives without being informative.

We chose to plot precision versus ranked gene pairs because when the data are graphed in this way, precision represents the fraction of gene pairs whose profile similarity is above a specified cut-off value that are co-annotated. This presentation seemed the most useful for choosing for future study non-co-annotated gene pairs that are likely to have a functional association. We also plotted the data in a more standard way as precision-recall curves. Recall, also known as sensitivity, is the fraction of real positives that a test identifies. It is equal to TP/(TP + FN), where True Positives + False Negatives is the number of real positives. We scored as True Positives gene pairs that are co-annotated in one or more annotation sets and whose profile similarity was above a specified cut-off value. Co-annotated gene pairs whose profile similarity was below the specified cut-off were scored as False Negatives. Precision and recall were calculated for the 5000 top-ranked gene pairs for each similarity metric. This cut-off was chosen because the low correlation values seen for gene pairs below the top 5000 are expected to be less useful in identifying functional associations. Supplementary Figure S7 shows precision-recall curves for gene pairs ranked based on either |PCC|, |SRCC|, or MI after minimal media conditions were excluded. This corresponds to the precision versus ranking graphs presented in Figure 4B. Both representations of the data show that highly correlated gene pairs were enriched for functional associations.

Precision-recall curves were also made that correspond to the precision versus ranking graphs shown in Figures 3 and 6. These are Supplementary Figures S6 and S8, respectively. The conclusions from these precision-recall curves are consistent with the conclusions made from the graphs of precision versus ranking.

Based on the precision scores for the top 500 ranked gene pairs, it initially appeared that |SRCC| and MI outperformed |PCC| (Figure 4A). However, when phenotypic profile similarity was recalculated after removing conditions involving growth on minimal media, the precision for gene pairs ranked based on |PCC| increased significantly, and there was now little difference in the performance of |PCC|, |SRCC| or MI (compare Figure 4, A and B). We suggest that this observed increase in precision for gene pairs ranked by |PCC| might be due to the sensitivity of the PCC to outliers in the data (Schober *et al.* 2018). We realized that the collection of strains used by Nichols *et al.* contains many mutants that have little or no growth on minimal media because the gene for a biosynthetic enzyme is deleted. Precision was low when minimal media growth conditions were included because so many combinations of genes from different biosynthetic pathways shared large, negative fitness scores on the 10 conditions involving minimal media but did not share a functional annotation in the annotation sets used. In general, the auxotrophic mutants did not have a significant phenotype in most of the other 314 growth conditions tested, which used rich media, so the large negative fitness scores on minimal media were essentially outliers. When these outliers were excluded, precision increased for the gene-pairs ranked based on |PCC|. We suggest that when high-throughput phenotype studies include conditions that involve defined media, such as testing for utilization of carbon or nitrogen sources, it would be useful to supplement the base minimal media with amino acids, nucleosides, and enzyme co-factors to reduce the phenotypic clustering of mutant strains unable to synthesize these compounds.

The results presented in Figure 4, A and B show that when gene-pairs are ranked by similarity calculated using the metrics |SRCC| or |MI|, precision did not change very much when conditions involving minimal media were excluded. While this observation might indicate that |SRCC| or MI are more useful for determining phenotypic profile similarity in high-throughput studies, we think it is premature to draw this conclusion based on analysis of only one phenotypic dataset. Moreover, for gene pairs ranked by |PCC|, many of the gene pairs that were excluded by eliminating the minimal media growth conditions would have been recognized as true positives if the annotation sets included annotations to cellular processes such as amino acid biosynthesis or nucleotide biosynthesis in addition to the annotations to metabolic pathways for individual compounds.

We conclude that there is no single best way to measure phenotypic profile similarity, and suggest it may be advantageous to use more than one correlation metric to look for functional associations. When we compared the 10,000 top-ranked gene pairs identified using either |PCC| or |SRCC| with minimal media conditions excluded, we found that each metric identified gene pairs not identified by the other. There were 204 gene pairs with |PCC| ≥ 0.5000 that were not present among the top 10,000 gene pairs ranked based on Spearman ranked correlation, and 87 gene pairs with |SRCC| ≥ 0.5000 that were not present among the top 10,000 gene pairs ranked based on Pearson correlation.

We also found differences among the highly ranked gene pairs when we compared gene pairs ranked by |PCC| when minimal media growth conditions were included or excluded. For most gene pairs that did not include an auxotrophic mutant, the phenotypic profile similarity based on |PCC| changed very little when minimal media conditions were removed (data not shown). However, there were a few gene pairs where a possible functional association could have been missed if the minimal media conditions were not removed. We illustrate this with a gene pair where the functions of the gene products are known to have a functional association. The *exbD* and *fepA* genes are both needed for transport of ferric iron-enterobactin across the outer membrane (Noinaj *et al.* 2010). When profile similarity was calculated using the fitness scores for all conditions, |PCC| = 0.4773. After minimal media conditions were removed, |PCC| increased to 0.6204, a high enough correlation that this gene pair would be a reasonable candidate for future experiments to test the prediction.

To make it easier to compare results for the different similarity metrics, we have made the dataset from Nichols *et al.* available in a searchable, interactive format that allows queries for strains, conditions, and phenotypic profile similarity of gene pairs determined by |PCC| with all conditions, |PCC| with minimal media conditions excluded, |SRCC|, MI, and semantic similarity (org/wiki/index.php?title=Special:Ecolispecialpage).

The relationship between precision and ranking based on profile similarity shown in Figure 4B suggests that a shared function is known for most of the highly correlated gene pairs. To test this idea, we used a cut-off of |PCC| >0.75 to define highly correlated gene pairs and then manually examined the non-co-annotated gene pairs. If fitness scores for the growth conditions involving minimal media were excluded, there were only 10 non-co-annotated gene pairs (summarized in Table 2). We found functional associations that could explain the observed phenotypic profile similarity for 7 of the 10 gene pairs. In one case, the two genes (*dsbB* and *dsbA*) showed up as non-co-annotated because they are in a pathway that was not yet included in EcoCyc release 21.0. The other six gene pairs highlight some of the challenges of creating (and using) annotation, such as deciding where pathways start and end and determining appropriate levels of granularity. For example, the gene pairs *rfaF*(*waaF*)-*rfaE*(*hldE*) and *rfaF*(*waaF*)-*lpcA* (*gmhA*) are non-co-annotated, even though all three genes are required for synthesis of the lipid A-core oligosaccharide component of outer membrane lipopolysaccharide. The explanation is that *rfaF*(*waaF*) is annotated to the central assembly pathway for building the lipid-core oligosaccharide moiety, while *rfaE*(*hldE*) and *lpcA*(*gmhA*) are annotated to a branch pathway that builds one of the saccharide subunits of the core (Raetz and Whitfield 2002). The functional association between the three genes would have been revealed if we had included GO annotations, since all three genes are annotated to the GO term for the lipopolysaccharide core region biosynthetic process (GO:0009244).

**Table 2:** Non-co-annotated gene pairs with |PCC|>0.75

**Gene pair** ^a^	Known or predicted functional association
ECK0730-*pal*_ECK0725-*ybgC*^b^	Tol-Pal cell envelope complex (CPLX0-2201)
ECK0768-*uvrB*_ ECK2563-*recO*	DNA repair: recombinational repair (RECFOR-CPLX) and nucleotide excision repair (UVRABC-CPLX)
ECK1912-*uvrC*_ECK2563-*recO*	DNA repair: recombinational repair (RECFOR-CPLX) and nucleotide excision repair (UVRABC-CPLX)
ECK2901-*visC*(*ubiI*)_ECK3033-*yqiC*(*ubiK*)^c^	Ubiquinol-8 biosynthesis (PWY-6708)
ECK3610-*rfaF*(*waaF*)_ECK3042-*rfaE*(*hldE*)^d^	Superpathway of lipopolysaccharide biosynthesis (LPSSYN-PWY)
ECK3610-*rfaF*(*waaF*)_ECK0223-*lpcA*^d^	Superpathway of lipopolysaccharide biosynthesis (LPSSYN-PWY)
ECK3852-*dsbA*_ECK1173-*dsbB*	Periplasmic disulfide bond formation (PWY0-1599)^e^
ECK1544-*gnsB*_ECK2394-*gltX*	Unknown
ECK2066-*yegK*(*pphC*)_ECK0345-*mhpB*	Unknown
ECK3699-*mnmE*_ECK0050-*apaH*	Unknown

a The strain names are from Supplementary Table S2 of Nichols *et al.* (2011). Where the gene name has changed, the new gene name is included in parentheses.

b *ybgC* is in an operon that also includes the genes for three of the protein components of the Tol-Pal cell envelope complex.

c *ubiK* codes for an accessory protein required for efficient synthesis of ubiquinol-8 under aerobic conditions, but is not annotated as part the ubiquinol-8 biosynthesis pathway.

d *rfaE*(*hldE*) and *lpcA* are not annotated to the super pathway of lipopolysaccharide biosynthesis (LPSSYN-PWY)

e PWY0-1599 was not present in EcoCyc release 21.0.

We did not find a shared function for the last three non-co-annotated gene pairs. Given that so many of the other highly correlated gene pairs do share a function, it is possible that future experiments will uncover a shared function for these three gene pairs. However, it also possible that the observed phenotypic profile similarity is fortuitous, as we saw for mutants with an auxotrophic phenotype or mutants with increased sensitivity to DNA damage. For example, this may be the most likely explanation for the phenotypic similarity of the *mnmE* and *apaH* genes. Both are required for growth at pH 4.5 (Nichols *et al.* 2011, Vivijs *et al.* 2016), but appear to function independently. MnmE, partnered with MnmG, modifies 2-thiouridine residues in the wobble position of tRNA anticodons (Elseviers *et al.* 1984), while ApaH is a diadenosine tetraphosphatase (Guranowski *et al.* 1983) and mRNA decapping enzyme (Luciano *et al.* 2019). Both MnmE and ApaH are proposed to affect resistance to pH and other stresses through their effects on gene expression (Dedon and Begley 2014; Vivijs *et al.* 2016; Luciano *et al.* 2019).

A significant conclusion from this study is that functional associations can still be inferred from phenotypic profiles after quantitative fitness scores are converted to discretized, ternary scores. While some information was lost compared to using the original quantitative fitness scores, the precision based on the ternary fitness scores was much greater than for randomly ordered gene pairs (Figure 6). This result suggests that discretized, ternary scores could be used to combine quantitative phenotype information from different studies. Using discretized scores might also allow qualitative phenotype information, such as aspects of cell morphology, to be incorporated into phenotypic profiles along with discretized quantitative phenotype information. This approach would also allow information from phenotype annotations, available from databases such as PomBase, SGD, or OMPwiki, to be incorporated into phenotypic profiles. The phenotype annotations typically capture information in a discretized fashion and have previously been shown to be useful for inferring gene function (Hoehndorf *et al.* 2013; Ascensao *et al.* 2014).

The precision of the discretized data could be increased by partitioning the quantitative scores into a larger number of bins, as shown in Supplementary Figure S9. Precision increased incrementally as the number of bins was increased from 3 to 5 bins, from 5 to 7 bins and from 7 to 9 bins. However, because the results from many phenotypic studies are not amenable to being partitioned into a larger number of bins, we believe that using ternary scores will maximize the number of datasets that can be combined and allow more inferences about gene function to be made from phenotypic information.
